# Incidence of Refeeding Syndrome in Critically Ill Children With Nutritional Support

**DOI:** 10.3389/fped.2022.932290

**Published:** 2022-06-21

**Authors:** Stéphanie Blanc, Tajnja Vasileva, Lyvonne N. Tume, Florent Baudin, Carole Chessel Ford, Corinne Chaparro Jotterand, Frederic V. Valla

**Affiliations:** ^1^HES-SO Master, HES-SO University of Applied Sciences and Arts Western Switzerland, University of Lausanne, Lausanne, Switzerland; ^2^School of Health and Society, University of Salford, Salford, United Kingdom; ^3^Pediatric Intensive Care, Hospices Civils de Lyon, Lyon, France; ^4^Department of Nutrition and Dietetics, Geneva School of Health Sciences, HES-SO University of Applied Sciences and Arts Western Switzerland, Geneva, Switzerland

**Keywords:** refeeding syndrome (RFS), critically ill children, nutritional support, pediatric intensive care unit, hypophosphatemia, hypokalemia, hypomagnesemia, malnutrition

## Abstract

**Introduction:**

Early enteral nutrition is recommended for critically ill children, potentially exposing those who are undernourished to the risk of refeeding syndrome. However, data on its incidence is lacking, and the heterogeneity of diagnostic criteria and frequent electrolyte disorders in this population make its diagnosis complex. In 2020, the American Society for Parenteral and Enteral Nutrition (ASPEN) developed consensus recommendations for identifying patients at risk and with refeeding syndrome. These state that undernourished children are considered at risk of refeeding syndrome; those who develop one significant electrolyte disorder (decrease ≥ 10% in phosphorus, potassium, and/or magnesium) within the first five days of nutritional support, combined with a significant increase in energy intake, are considered to have refeeding syndrome. The aim of this study was to determine the incidence of refeeding syndrome according to the ASPEN definition in critically ill children on nutritional support.

**Materials and Methods:**

A secondary analysis of two prospective cohorts conducted in a tertiary pediatric intensive care unit in France was undertaken, and additional data were retrospectively collected. Children included were those (0–18 years) admitted to the pediatric intensive care unit with a minimum of one phosphorus, potassium, and/or magnesium assay and who received exclusive or supplemental nutritional support. Undernourished children (body mass index z-score < –2 standard deviations) were considered at risk of refeeding syndrome. The ASPEN critiera were used to identify those with probable refeeding syndrome.

**Results:**

A total of 1,261 children were included in the study, with 199 children (15.8%) classified as undernourished, who were at risk of refeeding syndrome. Of these, 93 children were identified as having probable refeeding syndrome, giving an overall incidence of 7.4%. The incidence rate among at-risk children was 46.7%. Most patients (58.1%) were classified as having severe refeeding syndrome.

**Conclusion:**

Refeeding syndrome remains difficult to diagnose in critically ill children, due to frequent confounding factors impacting electrolyte plasma levels. These findings suggest that refeeding syndrome incidence may be high in undernourished children, and that refeeding syndromes can be severe. Further prospective studies using the ASPEN definition and risk criteria are required.

## Introduction

Refeeding syndrome (RS) is an acute metabolic disturbance that occurs upon reintroduction of oral, enteral nutrition (EN), or parenteral nutrition (PN) after prolonged fasting or suboptimal feeding (1). The body transitions from a catabolic to an anabolic state. This results in intracellular demand for inorganic phosphorus (P), potassium (K), magnesium (Mg), and thiamine (vitamin B1), causing hypophosphatemia, hypokalemia, and/or hypomagnesemia (1–3). RS can manifest itself as a mild electrolyte disorder with no associated clinical symptoms or as a severe electrolyte disorder leading, without supplementation, to severe organ failure, such as respiratory and cardiac failure, musculoskeletal weakness, and Wernicke’s encephalopathy (4–6). The mortality associated with RS ranges from 0 to 71% (7). In the pediatric intensive care unit (PICU), a mortality rate of 6% was observed among severely malnourished South African children who developed RS (8).

Children at risk of developing RS are those who have significantly reduced their energy intake for seven to ten days before the reintroduction of nutrition, and those who are undernourished (4–6, 9). The PICU population is thus at risk of RS at the time of refeeding (4), considering the high prevalence of malnutrition in this population, ranging from 15–25% (10–14), and the recommendation of introducing early EN within 24 hours of PICU admission (15).

The lack of consensus diagnostic criteria and the frequency of electrolyte disorders not associated with diet makes the diagnosis of RS complex in critically ill children (9). Therefore, data regarding its incidence is lacking. To our knowledge, only the American study by Dunn et al. (1999), conducted in children receiving PN in intermediate care and in the PICU, showed that 9% of the children included were at risk of RS but did not provide a clear incidence of RS (16). The authors described an electrolyte drop in 27% of subjects one day after PN, the most common being hypophosphatemia (16).

In March 2020, the American Society for Parenteral and Enteral Nutrition (ASPEN) published a consensus recommendation on, among other things, diagnostic criteria for RS for adults and children (4). These were: “*A decrease in any 1, 2, or 3 of serum phosphorus, potassium, or magnesium levels by 10%-20% (mild RS), 20%-30% (moderate RS), or* > *30% (severe RS), and/or organ dysfunction resulting from a decrease in any of these and/or due to thiamin deficiency (severe RS); And occurring within 5 days of reinitiating or substantially increasing energy provision*” (without specifying what a significant increase is).

The consequences of refeeding syndrome can be serious in critically ill children, who are a vulnerable population. Therefore, the main aim of this study was to measure the incidence of RS according to the ASPEN definition in critically ill children with nutritional support (NS), hospitalized in the PICU. The secondary objectives were to define the degree of severity of RS and to compare children with RS to others.

## Materials and Methods

### Study Design

A secondary analysis of two prospective enrolled cohorts was conducted and additional data were retrospectively collected. The data for these cohorts were collected from the PICU of the Hospices civils de Lyon (23-bed unit) in France, from September 2012 to August 2013 and September 2013 to December 2015 [Valla et al. (10, 17)].

### Study Subjects

Eligible children (0–18 years) were those included in the two prospective cohorts [Valla et al. (10, 17)], admitted to the PICU with a minimum of one P, K, and/or Mg assay, and who had received exclusive or supplemental NS by EN and/or PN during their stay. Children admitted to the PICU, but without assay of P, K or Mg or with oral exclusive nutrition were excluded. Local ethical clearance was obtained from the Hospices Civils de Lyon Ethics Committee (N° 21_433, 02/09/2021).

### Study Setting

This unit admitted children (0–18 years) with a variety of pathologies (trauma, infectious diseases, hematological diseases, surgery, and liver and kidney transplants), but not children with cardiac pathology and premature infants. Local nutrition guidelines included early EN, energy target (i.e., resting energy expenditure determined by Schofield equations) to be reached within three to five days, and nutrition prescriptions were monitored daily by a dietician. Plasma electrolyte level monitoring was undertaken according to patient’s clinical condition. In case of RS risk or RS occurrence identified by the PICU team, the local recommendations based on the guidelines of Melchior et al. (18) were as follows: thiamine, P, K, and Mg supplementation initiated at the start of refeeding. Blood electrolytes (P, K, Mg) were checked regularly during the first three days of refeeding and then daily between the fourth and eighth days (see reference (17) for management of nutritional support).

### Identification of Patients Who Met ASPEN Criteria for RS

**FIGURE 1:**
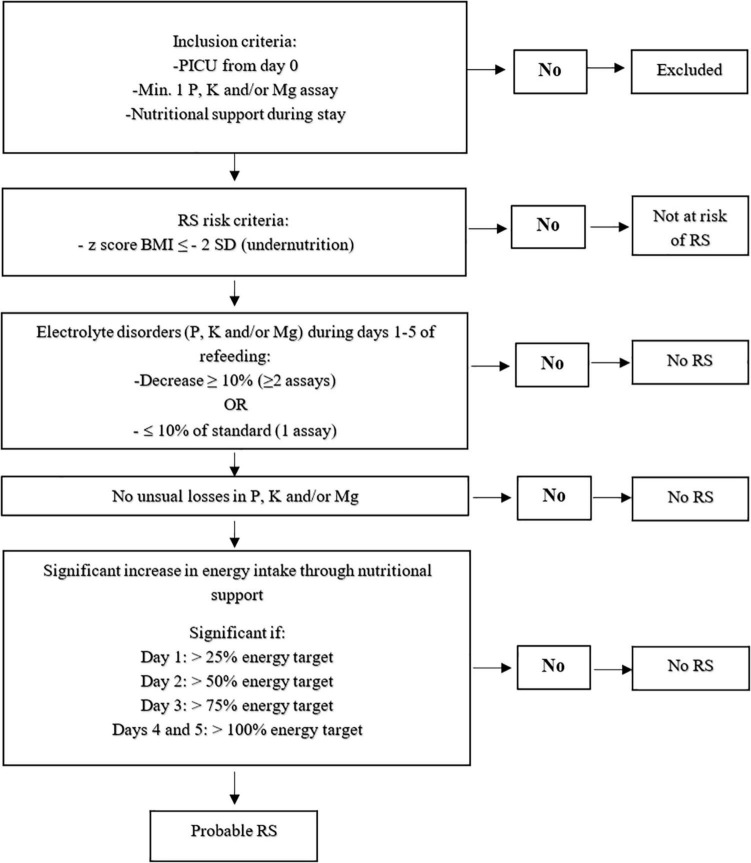
Refeeding syndrome assessment methodology. BMI = Body Mass Index, K = Potassium, Mg = Magnesium, P = Phosphorus, PICU = Pediatric Intensive Care Unit, RS = Refeeding Syndrome, SD = Standard Deviation.

The ASPEN criteria (4) were used to identify patients with probable RS, which are illustrated in Figure 1. The identification steps, in chronological order, were applied to children who met the inclusion criteria: (i) being at risk for RS, i.e., children undernourished at PICU admission (4, 6), then (ii) having presented with at least one significant electrolyte disturbance, i.e., decrease ≥ 10% in P, K, and/or Mg during the first five days of refeeding (days 1–5), and finally (iii) receiving energy intake that significantly increased during five days (days 1–5).

The severity of RS was also determined according to the ASPEN criteria (4): a reduction in serum P, K, and/or Mg levels between 10–20% corresponded to mild RS; between 20–30% to moderate RS; and greater than 30% to severe RS.

#### Assessment of Undernutrition

Undernutrition was considered when the Body Mass Index (BMI) z-score was ≤ –2 standard deviations (SD) (19). Although the BMI z-score is not the gold standard for assessing the nutritional status of children under two years of age (20), it is the only nutritional index available for which the World Health Organization (WHO) provides SD value for all ages of life as WHO does not provide data for the weight-for-height z-score for children older than five years (21). Anthropometric data were collected prospectively by the nutrition team during the studies of Valla et al. (10, 17) through a pre-established protocol and using recommended measurement tools.

#### Assessment of Significant Electrolyte Disorders

A decrease of ≥ 10% in P, K, and/or Mg was considered significant, according to the ASPEN definition (4). Electrolyte abnormalities were calculated using the difference between the first value before NS and the minimum value during the first five days of refeeding. In case of a single value during the PICU stay, this was compared to the lab lower normal value according to age (norms K: 3.5–5 mmol/l, P (≤ 18 months): 1.3–1.9 mmol/l, P (> 18 months): 0.8–1.3 mmol/l, Mg: 0.75–1.1 mmol/l). Children with electrolyte disorders who were diagnosed with unusual P, K, and/or Mg losses were not considered to have probable RS, as these diagnoses distorted the attribution of the electrolyte disorder to probable RS. These include tubulopathy, chronic renal failure, dialysis, renal transplants, extensive burns, epidermal necrolysis, Lyell’s syndrome, chronic enteropathy, significant vomiting, acute gastroenteritis, diabetic ketoacidosis, dysparathyroidism, and hyper/hypovitaminosis (6, 22).

#### Assessment of Significant Energy Intake

Daily energy intake during the first five days of refeeding was collected *via* electronic patient records. These included NS, that is, breast milk, infant formula, EN or PN (commercial formula or individualized) solutions, enrichment, glucose and propofol infusions. The volume of each solution administered and the nutritional compositions indicated by the manufacturers were used to calculate the daily energy intake of the patients. Breast milk intake was defined as 0.7 kcal/ml (23). Oral intake could not be included as this was not recorded.

Energy requirements were estimated using Schofield’s predictive equation with the child’s admission weight (24). As recommended (25), no correction factor was applied for children on mechanical ventilation (MV), as their energy expenditure at the PICU corresponds to their resting energy expenditure (25). For children on non-invasive ventilation (NIV) or non-ventilated children, a correction factor of 1.3 was applied according to the guidelines (15, 26).

ASPEN does not define what constitutes a significant increase in energy intake that may lead to RS (4). Based on the study by Van Zanten et al. (27) and according to the RS management protocol at the Hospices civils de Lyon, energy intake was considered significant if it was greater than 25% of the energy target on day 1, 50% of the energy target on day 2, 75% of the energy target on day 3, and 100% of the energy target on days 4 and 5.

#### Assessment of Confounding Factors

Confounding factors that may influence phosphatemia, kalemia, and/or magnesemia (6, 22) were also extracted. These included vomiting (days 1–5) and medications (days 1–5) such as insulin, diuretics, salbutamol^®^, chelators, antacids, catecholamines, steroids, and immunosuppressants.

## Statistical Analysis

The primary outcome of the study was the incidence of RS among critically ill children with NS admitted to the PICU. We decided to measure two incidences: one among the overall sample and one among the undernourished children. The number of children at each stage of the RS assessment is expressed as absolute (n) and relative (%) frequencies. The incidence of RS was calculated using the following formulas, with the results expressed as relative frequencies (%):

-Number of children with probable RS divided by the number of children in the baseline sample.

-Number of children with probable RS divided by the number of children at risk for RS (BMI z-score ≤ –2 SD).

Secondary outcomes were to describe children with probable RS and to compare them to all children without RS and to children at risk who did not developed RS. The following data were extracted from the studies by Valla et al. (10, 17) or from electronic patient records and compared among groups: child characteristics (age, sex, type and diagnosis of admission, Pediatric Logistic Organ Dysfunction I (PELOD I) and Pediatric Index of Mortality Score II (PIM II) severity scores (10, 17), respiratory support (duration of invasive ventilation and non-invasive ventilation), length of stay, acquired infections as defined by the Center for Disease Control (10) and mortality), electrolyte determination, nutritional support including supplementation (P, K, and/or Mg), and confounding factors.

To describe children with probable RS, categorical variables (including levels of RS severity) were expressed as absolute (n) and relative (%) frequencies. The distribution of quantitative variables was assessed by means of their mean, standard deviation, median, interquartile range, skewness, kurtosis, histogram, and box plot. Because the quantitative variables were not normally distributed, they were described by their median (p50) and interquartile range (Q1–Q3).

To compare children with probable RS to at-risk children who did not develop RS, the chi-square statistical test of homogeneity was used for categorical variables. However, no inference could be made on the categorical variables, which were too small to meet the conditions for the application of this statistical test. Quantitative variables were compared between the groups using the non-parametric Wilcoxon Mann-Whitney rank sum test. No statistical tests could be performed to compare children with probable RS and children without RS, because the sample size was so imbalanced between these two groups.

Statistical tests were conducted in a two-sided manner and the significance level was set at 5% (*p* < 0.05).

Statistical analyses were performed using StataIC^®^ version 16 software (StataCorp, College Station, TX, United States).

## Results

### Patients Identified as at Risk of RS or With RS, Incidence, and Severity Levels

**FIGURE 2:**
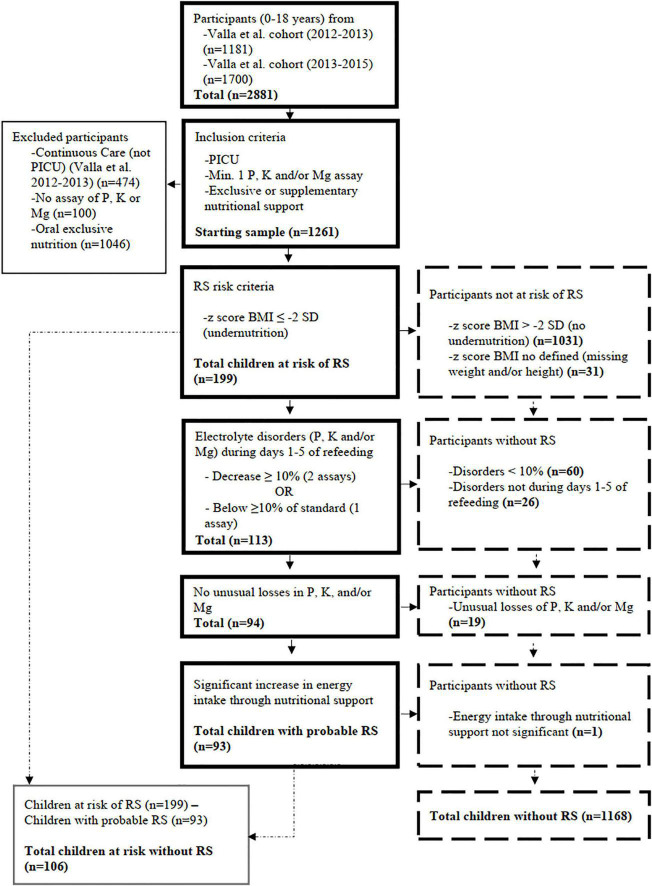
Refeeding syndrome assessment results. BMI = Body Mass Index, K = Potassium, Mg = Magnesium, P = Phosphorus, PICU = Pediatric Intensive Care Unit, RS = Refeeding Syndrome, SD = Standard Deviation.

The inclusion criteria were met by 1,261 children who constituted the baseline sample. A total of 199 children (15.8%) were undernourished (BMI z-score ≤ –2SD) and considered to be at risk for RS. Of these, 93 children probably developed RS. The flow of participants is shown in Figure 2.

The incidence of RS among critically ill children with NS was 7.4%. The incidence of RS among children at greater risk for developing RS (n = 199) was 46.7%. Among the 93 children with probable RS, 54 (58.1%) had severe RS, 21 (22.6%) had mild RS, and 18 (19.3%) had moderate RS.

## Patient Characteristics

The characteristics of the participants are described in Table 1. Children were classified into three groups: those who developed probable RS (“RS child”), those without RS (“without RS”), and those who were at risk of RS but who did not developed RS (“at RS risk”). The PELOD I and PIM II scores were significantly higher in “RS child” than in the “at RS risk” (*p* = 0.003 and *p* = 0.011). The length of PICU stay was the longest in “RS child” than in “at RS risk” (*p* < 0.001). The frequency of acquired infection during the hospital stay was also the highest in “RS child” than in “at RS risk” (*p* = 0.002). Compared to the “at RS risk” group, “RS child” were more frequently mechanically ventilated (*p* = 0.001).

**TABLE 1:** Group characteristics.

Characteristics	“RS child” Group 1	“Without RS” Group 2	“At RS risk” Group 3	Group 1 vs 3 *p*-value*
Participants (*n*)	93	1,168	106	
Age (month)	5.9 (2 – 59)	7.7 (1.5 – 58.5)	5.1 (2.1 – 46.9)	0.520
**Gender**				0.718
Girls	38 (40.9%)	483 (41.3%)	46 (43.4%)	
Boys	55 (59.1%)	685 (58.7%)	60 (56.6%)	
**Admission**				0.360
Surgical	17 (18.3%)	262 (22.4%)	25 (23.6%)	
Medical	76 (81.7%)	906 (77.6%)	81 (76.4%)	
**Diagnosis**				–
Respiratory failure	59 (63.4%)	683 (58.5%)	66 (62.3%)	
Neurology	15 (16.1%)	161 (13.8%)	8 (7.6%)	
Gastrointestinal	10 (10.8%)	106 (9.1%)	11 (10.3%)	
Sepsis	4 (4.3%)	63 (5.4%)	3 (2.8%)	
Hemodynamic	1 (1.1%)	39 (3.3%)	3 (2.8%)	
Nephrology	0 (0%)	43 (3.7%)	8 (7.6%)	
Trauma	1 (1.1%)	38 (3.2%)	0 (0%)	
Other	3 (3.2%)	35 (3%)	7 (6.6%)	
PELOD score	11 (11 – 13)	11 (8 – 12)	11 (10 – 11)	**0.003**
PIM II score	5.8 (2 – 12.4)	4 (1.3 – 7.9)	3.1 (1.2 – 8.2)	**0.011**
Length of stay (*days*)	9 (5.8 – 15.2)	6 (3.6 – 10.8)	4.6 (2.9 – 9.4)	** < 0.001**
Mechanical ventilation (MV)	63 (67.7%)	612 (52.4%)	46 (43.4%)	**0.001**
MV duration (*days*)	5 (2 – 12)	4 (2 – 9)	2 (1 – 9)	**0.010**
Non-invasive ventilation (NIV)	68 (73.1%)	696 (59.6%)	66 (62.3%)	0.103
NIV duration (*days*)	4 (1.5 – 11.5)	3 (2 – 7)	3 (2 – 7)	0.419
Without ventilation	3 (3.2%)	115 (9.9%)	15 (14.2%)	–
Acquired infection	30 (32.3%)	260 (22.3%)	15 (14.2%)	**0.002**
Death	6 (6.5%)	51 (4.4%)	2 (1.9%)	–
Admission weight (*kg*)	6 (3.3 – 15)	7.6 (4 – 18)	4.7 (3.2 – 11)	0.845
Admission height (*cm*)	65.5 (51 – 115)	66 (52.5 – 104)	60.5 (51.5 – 100)	0.905
z score BMI (*SD*)	−2.8 (−3.4 – −2.3)	−0.3 (−1.2 – 0.8)	−2.6 (−3.4 – −2.3)	0.353
z score height/age (*SD*)	−1.5 (−4 – −0.1)	−0.7 (−2 – 0.6)	−1 (−2.6 – 0.6)	0.123

*“RS child” are the children with probable Refeeding Syndrome, “without RS” are all the children without Refeeding Syndrome, “at RS risk” are the children who were considered at risk of Refeeding Syndrome because they were undernourished but who didn’t develop it.*

*Values are presented as absolute (n) and relative (%) frequencies or median and IQR (Q1–Q3).*

*Significant p values < 0.05 are shown in bold.

*“–” means that no statistical test could be applied.*

*BMI = Body Mass Index, PELOD = Pediatric Logistic Organ Dysfunction, PIM II = Pediatric Index of Mortality II, RS = Refeeding Syndrome, SD = Standard Deviation.*

## Electrolyte Disorders

The frequency of significant electrolyte disorders (P, K and/or Mg) was 67.3% in the baseline sample (see Supplementary Figure 1).

**FIGURE 3:**
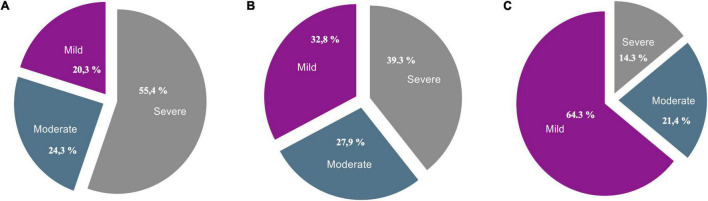
Frequencies [absolute (*n*) and relative (%)] of severity levels of phosphorus **(A)** potassium **(B)** and magnesium **(C)** disorders, within the children with probable Refeeding Syndrome. **(A)** Total (*n* = 74), Mild (*n* = 15), Moderate (*n* = 18), Severe (*n* = 41). **(B)** Total (*n* = 61), Mild (*n* = 20), Moderate (*n* = 17), Severe (*n* = 24). **(C)** Total (*n* = 14), Mild (*n* = 9), Moderate (*n* = 3), Severe (*n* = 2).

Forty-four children (47.3%) of “RS child” had a significant disorder in one of the three electrolytes (P, K, or Mg), 42 (45.1%) in two electrolytes, and 7 (7.6%) in all. Seventy-four children (79.6%) had a significant phosphate disorder, the majority of which was severe (55.4%). A total of 61 children (65.6%) had a significant potassium disorder, the majority of which was also severe (39.3%). Fourteen children (15.1%) had a significant Mg disorder, the majority of which were mild (64.3%) (Figure 3).

**FIGURE 4:**
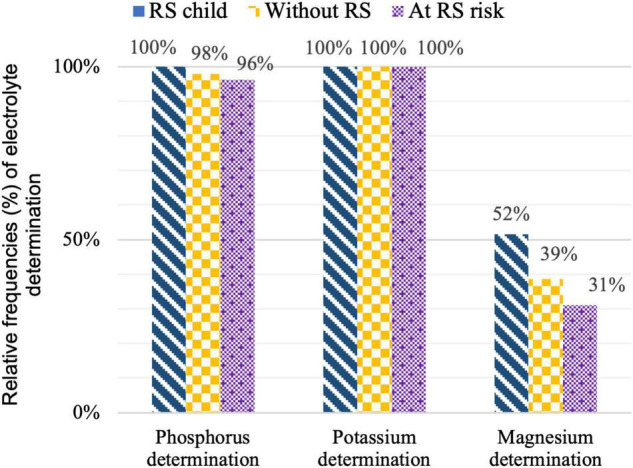
Relative frequencies (%) of electrolyte determination within the children with probable Refeeding Syndrome (“RS child”), these without Refeeding Syndrome (“without RS”) and these at Refeeding Syndrome Risk (“at RS risk”).

The percentage of electrolyte disorders according to the number of determinations in children with probable RS (“RS child”) is presented in Table 2. Figure 4 shows the frequency of electrolyte determination by group. In “RS child,” Mg was measured more often than “at RS risk” (*p* = 0.005).

**TABLE 2:** Electrolyte disorders of phosphorus, potassium and magnesium in the children identified with a probable Refeeding Syndrome.

Electrolyte disorders	“RS child” *n* (%)	p50 (Q1 – Q3)
**Significant decrease between two values (days 1–5)**		
Phosphorus	71 (76.3%)	31.6% (21.8 – 44.1)
Magnesium	10 (10.8%)	17.8% (13.4 – 21.5)
Potassium	61 (65.6%)	25.6% (17.5 – 34)
**Significant disorder according to the standard (one value) (days 1–5)**		
Phosphorus	3 (3.2%)	26.3% (21.5 – 33.8)
Magnesium	4 (4.3%)	23.3% (16 – 33.3)
Potassium	0 (0%)	

*The children with probable Refeeding Syndrome correspond to “RS child” in the table.*

*The frequency of children who presented a disorder for each electrolyte are presented in absolute (n) and relative (%) frequencies.*

*Mediane (p50) and IQR describe the electrolyte decrease (in %) between two values or the difference from the standard value (in %).*

## Nutritional Intake

The types of nutritional support and supplementation provided to the children in the different groups are shown in Table 3. Most children were enterally fed (91.8%) and few received parenteral nutrition (15.8%). “RS child” received PN more frequently than “at RS risk” (*p* = 0.019). They also received a combination of EN and PN more frequently than “at RS risk” (*p* = 0.023). They received more supplementation with electrolytes than “at RS risk” (*p* = 0.001), mainly with P (*p* = 0.001).

**TABLE 3:** Nutritional support and electrolyte supplementation in the three groups.

Nutritional support and supplementation	“RS child” Group 1	“Without RS” Group 2	“At RS risk” Group 3	Group 1 vs 3 *p*-value*
Participants (*n*)	93	1,168	106	
Enteral nutrition (EN) EN	85 (91.4%) 72 (77.4%) 13 (14%)	1,081 (92.6%) 989 (84.8%) 92 (7.8%)	100 (94.3%) 95 (89.6%) 5 (4.7%)	0.418
EN + PN				**0.023**
Parenteral (PN) PN	21 (22.6%) 8 (8.6%)	179 (15.2%) 87 (7.4%)	11 (10.4%) 6 (5.7%)	**0.019**
PN + EN	13 (14%)	92 (7.8%)	5 (4.7%)	**0.023**
Electrolyte supplementation (P, K, Mg) (days 1–5) Number of electrolyte supplementation	30 (32%)	166 (14.2%)	9 (8.5%)	**< 0.001**
1 supplémentation	28 (30.1%)	139 (11.9%)	9 (8.5%)	–
2 supplémentations	2 (2.1%)	25 (2.1%)	0 (0%)	
3 supplémentations	0 (0%)	2 (0.2%)	0 (0%)	
P supplementation (days 1–5)	24 (25.8%)	126 (10.8%)	9 (8.5%)	**0.001**
K supplementation (days 1–5)	8 (8.6%)	56 (4.8%)	0 (0%)	–
Mgsupplementation (days 1–5)	0 (0%)	13 (1.1%)	0 (0%)	–

*“RS child” are the children with probable Refeeding Syndrome, “without RS” are all the children without Refeeding Syndrome, “at RS risk” are the children who were considered at risk of Refeeding Syndrome because they were undernourished but who didn’t develop it.*

*Values are presented as absolute (n) and relative (%) frequencies.*

*Significant p values < 0.05 are shown in bold.

*“–” means that no statistical test could be applied.*

*K = Potassium, Mg = Magnesium, P = Phosphorus.*

**FIGURE 5:**
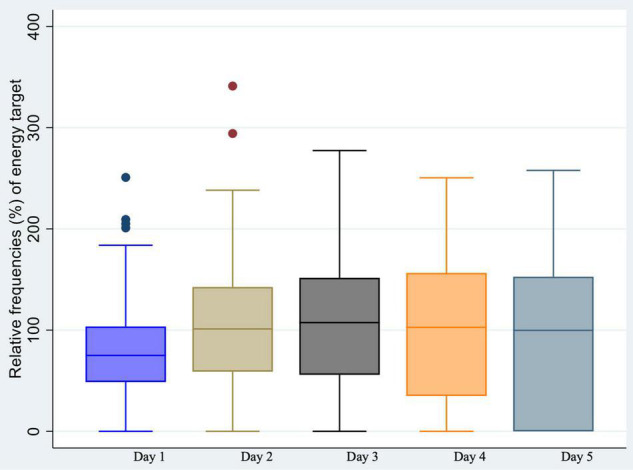
Daily energy intake *via* nutritional support expressed as relative frequencies (%) of the energy target, within the children with probable Refeeding Syndrome.

**FIGURE 6:**
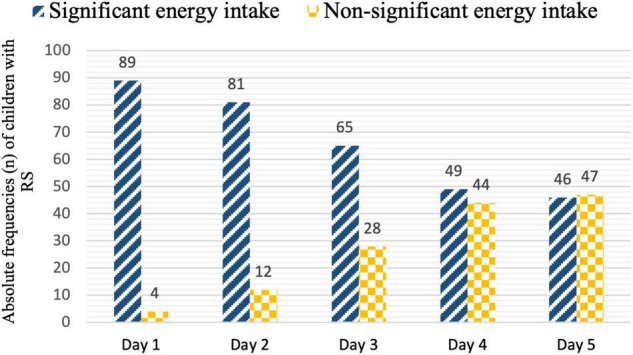
Absolute frequencies (*n*) of significant versus non-significant daily intakes within the children with Refeeding Syndrome (RS) (*n* = 93). Significant intakes are considered: Day 1 intakes > 25% of energy target, Day 2 intakes > 50% of energy target, Day 3 intakes > 75% of energy target, Days 4 and 5 intakes > 100% of energy target.

Energy intake *via* NS for “RS child,” expressed as a percentage of the energy target, is shown for each day in Figure 5. The energy target was already covered by day 2 (101.2%; IQR:58.9-142.5). Significant energy intake are described in Figure 6.

## Confounding Factors

The frequencies of confounding factors for the children in the three groups are shown in Table 4. Most children had two and more confounding factors in “RS child” group, while many children in “at RS risk” had none or one confounding factors (*p* = 0.041). The frequency of diuretic use was also the highest in “RS child” (“at RS risk,” *p* < 0.001).

**TABLE 4:** Confounding factors in the three groups.

Confounding factors (days 1–5)	“RS child” Group 1	“Without RS” Group 2	“At RS risk” Group 3	Group 1 *VS* 3 *p*-value*
Participants (*n*)	93	1,168	106	
Confounding factors frequencies	23 (24.7%)	358 (30.6%)	29 (27%)	**0.041**
None				
1	18 (19.4%)	342 (29.3%)	35 (33%)	
≥ 2	52 (55.9%)	468 (40.1%)	42 (40%)	
Vomiting	30 (32.3%)	365 (31.3%)	41 (38.7%)	0.345
Insulin	3 (3.2%)	18 (1.5%)	2 (1.9%)	–
Chelators	1 (1.1%)	14 (1.2%)	1 (0.9%)	–
Diuretics	46 (49.5%)	308 (26.4%)	18 (17%)	** < 0.001**
Immunosuppressants	3 (3.3%)	52 (4.5%)	2 (1.9%)	–
Salbutamol	17 (18.3%)	163 (14%)	15 (14.2%)	0.429
Steroids	22 (23.7%)	236 (20.2%)	22 (20.8%)	0.623
Antacids	21 (22.6%)	285 (24.4%)	30 (28.3%)	0.356
Catecholamines	24 (25.8%)	219 (18.8%)	17 (16%)	0.089

*“RS child” are the children with probable Refeeding Syndrome, “without RS” are all the children without Refeeding Syndrome, “at RS risk” are the children who were considered at risk of Refeeding Syndrome because they were undernourished but who didn’t develop it.*

*Values are presented as absolute (n) and relative (%) frequencies.*

*Significant p values < 0.05 are shown in bold.

*“–” means that no statistical test could be applied.*

## Discussion

### Refeeding Syndrome Incidence and Severity Levels

The incidence of RS was high: 7.4% among children admitted to the PICU with NS and 46.7% among at-risk children (BMI z-score ≤ –2SD). The results of this study are difficult to compare with those of other studies, as the population (including age, pathology, type of NS, and care units) and criteria used to define RS are heterogeneous in the available literature. In 1999, Dunn et al. showed that 9% of children receiving PN in continuous care were at risk of RS but did not provide a clear incidence of RS (16). Recently, a systematic review by Bradford et al. (28) concluded that the incidence of RS in neonatology based on electrolyte disorders of P, K, and Mg is unknown, with most studies assessing hypophosphatemia only. Other studies have reported incidences of 0% (29), 14% (30), 15% (8), 19% (31), 23% (32), and 60% (33), respectively, but none of these studies were conducted in PICUs. The results highlight the complexity of identifying RS in critically ill children for several reasons.

In the baseline sample, the frequency of significant electrolyte disturbances was high (67.3%). This rate is consistent with previous PICU data i.e., 5–50% hypophosphatemia (22), 40% hypokalemia (34) and > 50% hypomagnesemia (35). In addition, children with probable RS had more confounding factors, including more diuretics, than others. Thus, it is possible that the electrolyte disturbances identified may have been more associated with diuretics than with diet, overestimating the incidence rate of RS.

However, other factors may have underestimated the incidence of RS. In the current study, the assessment of being at risk of RS was based on the BMI z-score, a static assessment that was available for all included children. Children presenting with recent weight loss (dynamic assessment of nutritional status) may also be considered at risk of RS. Furthermore, due to the retrospective nature of this study, we could not use the full ASPEN diagnostic criteria (4) i.e., organ dysfunction resulting from a decrease in any of P, K or Mg and/or due to thiamin deficiency (severe RS). It is possible that using these criteria would have led to a higher rate of RS in our cohort. In addition, the lack of routine electrolyte determinations (P, K, and Mg not systematically included in the entry serum electrolytes before the start of NS and then daily between days 1–5) also decreased the likelihood of diagnosing RS. Additionally, children with unusual P, K, and/or Mg losses were excluded, but they may also have presented with RS.

Most of the RS identified in our study were classified as severe. Significant energy intakes during the first five days of refeeding may explain this severity. This result also appeared to be consistent with a recent study by Schalpfer et al. (36), which showed that children had severe RS according to the ASPEN criteria.

## Comparison of Children With RS

When we compared the children with probable RS with the “at RS risk,” we did not observe any significant differences in age, gender or reason for admission to the PICU. However, we observed that they had a longer PICU length of stay. The latter was almost 50% longer than that of children at risk of RS. This was consistent with the fact that they had factors that could impact their PICU length of stay: they had significantly higher severity scores, were more frequently on mechanical ventilation, and had higher rates of acquired infections. This is consistent with the ASPEN (4) which defines acquired immunodeficency syndrome as a clinical condition associated with an increased risk of RS.

The mortality rate in children with probable RS was 6.5%. This was consistent with the study by Mbethe and Mda (8), which found a 6% mortality rate among severely malnourished children in the PICU who developed RS. In this study, we were not able to statistically compare the results with those of the children at risk of RS due to small sample size.

Most of the children in our study were enterally fed (91.4–94.4%). This figure is in line with guidelines advocating that EN should be preferred in critically ill children (37). Regarding the management of NS for children with probable RS, the median energy intake on day 1 was high (75% of the energy target), and the energy target was already covered by day 2 (101.2%). For almost all children (95.7%), intake at day 1 was considered significant according to our criteria (> 25% of the energy target). These results could partly explain the incidence of RS, and the severity of cases. However, the protocol of RS management of the Hospices Civils de Lyon reached the energy target after three to five days of refeeding. Several factors could explain this discrepancy. Children with RS were more likely to receive a combination of EN and PN than others. The addition of PN during the first five days significantly increases energy intake (37) and may increase the risk of overfeeding. However, there is no consensus regarding the energy target during the first days of refeeding (5). The ASPEN recommends that energy intake should not exceed 40–50% of the target (4). Others have described an intake of 20–75% of the target energy during the first few days of refeeding as “safe” (1, 5) (i.e., a range of one to more than three times). Therefore, the criteria chosen in this study to define significant energy intake were strict compared to others. Finally, children with probable RS were supplemented more than others with P, K, and/or Mg, which is consistent with the protocol for identifying and correcting electrolyte disorders.

### Limitations and Strengths

The study is limited by its retrospective design, not allowing collecting oral intake data and using the ASPEN diagnostic criteria for organ dysfunction resulting from decreased electrolyte and/or thiamine deficiency (4). The use of the sole BMI z-score (static assessment) to define nutritional status could lead to a misclassification bias in undernourished children and, consequently, in children assessed with probable RS. However, the prevalence of undernutrition using the BMI z-score as a diagnostic criterion was 15.8%, which is consistent with previous PICU data i.e., 15–25% (10–14). To avoid excluding children with only one electrolyte measurement during the PICU stay, a lower age standard was used to calculate serum level reduction. This choice may have underestimated the electrolyte disorder (the actual value before the start of NS may have been higher than the normal age value), conversely, it may also have been overestimated (the child may have been admitted with an already depleted normal value). This may have resulted in a misclassification bias in children with significant electrolyte disorders. However, this bias affected a minority of children among those identified with significant electrolyte disturbances in the baseline sample (41 children out of the 849 children assessed with a significant electrolyte disturbance). During the time of this study, the pepanic study results (suggesting benefit from late PN) (38) were not published, and PN was started before day 7 in the case of EN contraindication or as supplemental PN. However, PN adjunction was prescribed similarly to EN in order to meet energy targets within three to five days, which should not have increased the likelihood of presenting with RS. Finally, it would have been useful to calculate the energy intake of all children in order to compare them between groups but this was not planned in the study design.

To our knowledge, this is the first study to measure the incidence of RS in critically ill children, including all types of nutritional intakes, and based on the recent ASPEN consensus definition (4). This study also differentiated the incidence of RS among all included children and those at risk. Using the ASPEN definition, this study is the first to generate data on the severity levels of RS. The sample size was large and included participants from two prospective cohorts admitted to the PICU for over two years. This supports the external validity of this study. Anthropometric data were collected in a way that also favored their validity and reliability [see references (10, 17)]. In addition, the use of electronic patient records for daily energy intake (NS, glucose, and propofol), supplementation (P, K, and/or Mg), and confounding factors was reliable and reflected the quantities administered to the patients. The temporality between electrolyte disturbances and the first five days of refeeding could also be controlled to the hour within the electronic patient record. Finally, we had very minimal missing data.

## Conclusion

The incidence of RS among critically ill children at risk was high (46.7%), and severe most of the time. The PICU length of stay and acquired infections were greater in children who developed RS than in those at risk who did not. This underscores the importance of screening children for RS in the PICU, particularly in children with higher severity scores, in order to diagnose, prevent, treat (including thiamine supplementation) and monitor (with systematic P, Mg and K assays) RS adequately. Our study also revealed that the lack of consensus on precise refeeding recommendations may lead to excessive intake and may promote RS. This finding demonstrates the importance of establishing new progressive refeeding recommendations for the first few days after PICU admission. Finally, RS is difficult to assess in the PICU, and prospective studies using all ASPEN risks and diagnostic criteria are needed.

## Data Availability Statement

The raw data supporting the conclusions of this article will be made available by the authors, without undue reservation.

## Ethics Statement

The studies involving human participants were reviewed and approved by Scientific and Ethical Committee of Hospices Civils de Lyon, France. Written informed consent was obtained from the individual(s), and minor(s)’ legal guardian/next of kin, for the publication of any potentially identifiable images or data included in this article.

## Author Contributions

FV, CCJ, SB, and TV contributed to the study conception. FV and CCF recruited the patients and collected the data in previous studies [study references: (10, 18)]. FV and FB extracted additional data from electronic patient records. SB and TV calculated nutritional intake and performed the statistical analyses and analyzed the results in collaboration with FV and CCJ. SB, TV, FV, CCJ, and LT interpreted the data. LT edited the English. SB and TV wrote the draft manuscript, which was reviewed and approved by all the authors. All authors contributed to the article and approved the submitted version.

## Conflict of Interest

The authors declare that the research was conducted in the absence of any commercial or financial relationships that could be construed as a potential conflict of interest.

## Publisher’s Note

All claims expressed in this article are solely those of the authors and do not necessarily represent those of their affiliated organizations, or those of the publisher, the editors and the reviewers. Any product that may be evaluated in this article, or claim that may be made by its manufacturer, is not guaranteed or endorsed by the publisher.

## Abbreviations

ASPEN: American Society for Parenteral and Enteral Nutrition
EN: Enteral Nutrition
K: Potassium
Mg: Magnesium
NS: Nutritional Support
P: Phosphorus
PN: Parenteral Nutrition
PICU: Pediatric Intensive Care Unit
RS: Refeeding Syndrome.
