# Supplementary figures and images for: Incidence of Refeeding Syndrome in Critically Ill Children With Nutritional Support
Source: Front Pediatr. 2022 Jun 21;10:932290. doi: 10.3389/fped.2022.932290 (PMC9253668; doi:10.3389/fped.2022.932290)

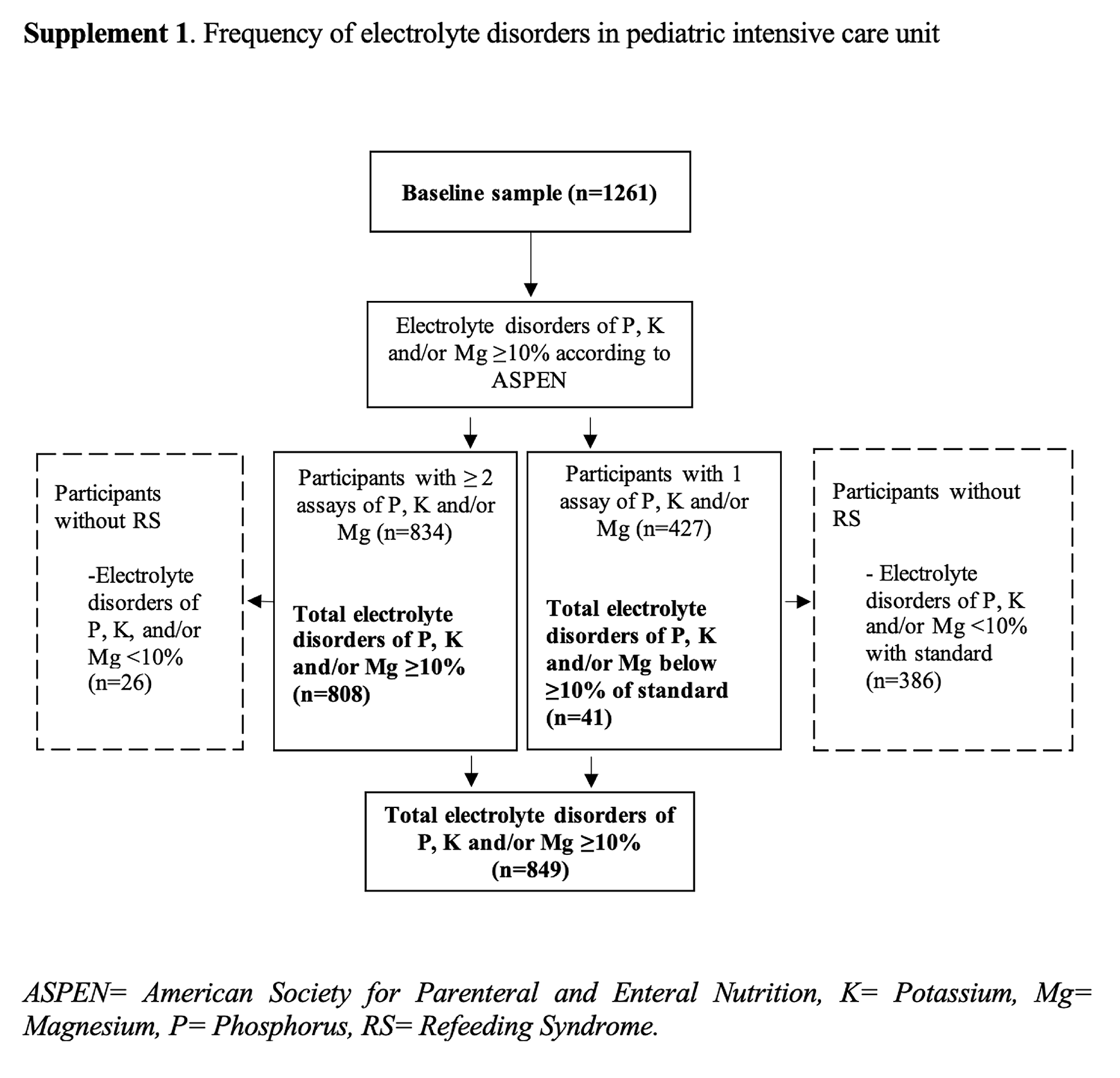

Supplement: Supplementary file 1 [file Image_1.TIFF]
